# A retrospective case series of segmental zoster paresis of limbs: clinical, electrophysiological and imaging characteristics

**DOI:** 10.1186/s12883-018-1130-4

**Published:** 2018-08-21

**Authors:** Ying Liu, Bing-Yun Wu, Zhen-Shen Ma, Juan-Juan Xu, Bing Yang, Heng Li, Rui-Sheng Duan

**Affiliations:** 1 Department of Electromyography, Shandong Provincial Qianfoshan Hospital, Shandong University, Jinan, 250014 People’s Republic of China; 2 Department of radiology, Shandong Provincial Qianfoshan Hospital, Shandong University, Jinan, 250014 People’s Republic of China; 3 Department of Neurology, Shandong Provincial Qianfoshan Hospital, Shandong University, Jinan, 250014 People’s Republic of China

**Keywords:** Herpes zoster, Segmental zoster paresis, Infectious neuropathy, Nerve conduction, Electromyography, Nerve MRI

## Abstract

**Background:**

Segmental zoster paresis (SZP) of limbs, characterized by focal weakness of extremity, is recognized as a rare complication of herpes zoster (HZ). The following study analyzes the clinical characteristics and data from electromyography and MRI scans in patients with motor weakness after zoster infection.

**Methods:**

One thousand three hundred ninety-three patients from our database (Shandong Provincial Qianfoshan Hospital) suffering from HZ were retrospectively reviewed from June 2015 to July 2017. Patients who fulfilled the diagnostic criteria for SZP were included in the analysis. The clinical characteristics, as well as electromyography findings and MRI scans were analyzed.

**Results:**

SZP was present in 0.57% of patients with HZ (8/1393). The average age of symptom onset in 8 SZP patients was 69 years old (SD: 13, range 47–87). The severity of muscle weakness ranged from mild to severe. The electrophysiological testing revealed the characteristics of axonopathy. Radiculopathy (2/8), plexopathy (2/8), radiculoplexopathy (3/8) and combined radiculopathy and mononeuropathy (1/8) were also identified. MRI revealed hyperintensity of the affected spinal dorsal horns, nerve roots or peripheral nerves.

**Conclusions:**

SZP is associated with obvious limb weakness, nerve axons lesions and localization to nerve roots, plexus or peripheral nerves.

## Background

Herpes zoster (HZ) is caused by varicella-zoster virus (VZV) which is latent in the dorsal root ganglia and reactivates when the immune system is not functioning properly. The incidence of HZ is about 4–4.5 per 1000 person-years [1], which is characterized by vesicular rash and burning pain. Postherpetic neuralgia is the most common neurologic syndrome of HZ, whereas segmental zoster paresis (SZP) of limbs is a relatively rare complication, characterized by focal weakness of upper or lower extremity. The motor involvement can be observed in 0.5–5% patients with HZ [2, 3].

The exact mechanism of SZP is not clear, although the spread of virus along the nerve is presumed [4–6]. The existing literature mainly includes case reports [7–18]. Comparatively, only few studies have described case series of SZP based on electrophysiological, imaging characteristics and prognosis data. In addition, antiviral medications and corticosteroids are the most commonly applied drugs for treating SZP. The prognosis for patients with SZP is generally favorable; however, in some extreme cases it can lead to permanent disability [2].

Here, we investigated clinical, electrophysiological and imaging evidence in a case series of 8 patients with SZP, as well as possible factors influencing prognosis.

## Methods

### Patients

The patient database in Shandong Provincial Qianfoshan Hospital was reviewed for coded diagnoses of HZ from June 2015 to July 2017. Patients with HZ were diagnosed as SZP based on the following criteria [4]: 1) Infection of HZ preceding or following limb paresis established based on historical or physical examination evidence of a cutaneous vesicular eruption; 2) geographical (same limb) and temporal (no more than 30 days) evidence of associated limb weakness which was confirmed by a neurologist; 3) the lesions of nerve roots and plexus or mononeuropathy associated with HZ which were verified using electrophysiological testing.

Weakness was identified in each affected muscle as mild (corresponding to MRC 4 or 4+), moderate (corresponding to MRC 3), severe (corresponding to MRC 2 or 1) and complete (corresponding to MRC 0).

According to the Medical Research Council scale, the muscle recovery was based on the following criteria: 1) complete recovery, if muscle strength was evaluated as grade 5 and the sensory symptom disappeared in the last follow-up visit; 2) no recovery, if no improvement in muscle strength was observed; 3) partial recovery, case between the complete and no recovery. In addition, all patients were followed up for approx. 0.5–2.0 years; the follow-up evaluation was based on sensorimotor symptom and muscle strength.

This retrospective study was approved by the medical ethics committee at our hospital.

### Electrophysiology

Nihon Kohden MEB-9400 electromyograph was used to evaluate nerve injury. Surface electrodes were used to perform nerve conduction studies (NCS), including motor and sensory nerve conduction velocity (NCV), distal motor latency (DML), amplitudes of compound muscle action potentials (CMAP_S_) and sensory nerve action potentials (SNAP_S_).

Median motor study was performed with distal stimulation site over the median nerve at the wrist and proximal stimulation site at the antecubital fossa, recording the abductor pollicis brevis muscle. Ulnar motor study was performed with distal stimulation site over the ulnar nerve at the wrist and proximal stimulation site above the elbow, recording the abductor digiti minimi muscle. Radial motor study was performed with distal stimulation site in the forearm and proximal stimulation site in the arm, below the spiral groove, recording the extensor indicis proprius muscle.

Tibial motor study was performed with distal stimulation site slightly proximal and posterior to the medial malleolus and proximal stimulation site in the middle of the popliteal fossa, recording the abductor hallucis brevis muscle. Peroneal motor study was performed with distal stimulation site over the anterior ankle and proximal stimulation site below the fibular head, recording the extensor digitorum brevis muscle.

Axillary, suprascapular, musculocutaneous motor conduction studies were performed, recording the deltoid, supraspinatus and biceps brachii with surface electrodes repectively, and stimulation site was at Erb’s point.

Antidromic and orthodromic sensory studies were performed in upper extremity or lower extremity respectively. Index finger and little finger were respectively stimulated to record SNAPs of the median and ulnar nerve.

A comparison with contralateral (asymptomatic) sides or normal values helped to determine the degree of damage. If NCV was slower than 75% of the lower limit of normal, and DML was longer than 130% of the upper limit of normal, it was regarded as demyelination. If the side-to-side amplitudes difference was greater than 50%, it was regarded as abnormal and axonal lesion.

The needle electromyography (EMG) was performed by a concentric needle electrode, which was used to find spontaneous potentials such as positive sharp waves and fibrillation potentials conventionally graded from 0 to 4+ as follows: 0 for none present; + 1 for persistent single trains of potentials (> 2–3 s) in at least two areas; + 2 for moderate number of potentials in three or more areas; + 3 for many potentials in all areas; + 4 for full interference pattern of potentials. The firing pattern (activation, recruitment and interference pattern) was assessed.

According to SNAP and spontaneous activities in paraspinal muscles, associated limb was characterized as preganglionic, postganglionic lesions or combined pre- and postganglionic lesions, and then further identified as radiculopathy, plexopathy, radiculoplexopathy and peripheral nerves [4] .

### Imaging

The MRI results were reviewed by an experienced radiologist. All studies included both T1- and T2- weighted images in coronal planes, without gadolinium-enhanced T1-weighted images. All T2-weighted sequences had fat saturation or a short tau inversion recovery sequence. Nerve root, plexus, or peripheral nerve images were identified as abnormal if prolonged nerve T2 or nerve enlargement was shown based on comparison with contralateral neural structures within the imaging field.

## Results

### Patient demographics and clinical characteristics

Eight out of 1393 inpatients with HZ fulfilled the diagnostic criteria for SZP, accounting for 0.57%, which was confirmed by neurologic examination and electrodiagnostic evaluation. Clinical characteristics are listed in Table 1. Detailed information of 8 patients are described in Table 2. Three descriptive cases are described in detail in the following paragraphs.

**Table 1:** Demographics and clinical characteristics in patients with segmental zoster paresis

Features	
Mean age (years)	69 (range 47–87)
Men	3 of 8
Affected myotomes	
Left upper limb	1 of 8
Right upper limb	5 of 8
Left lower limb	0 of 8
Right lower limb	2 of 8
Rash after weakness (within 30 days)	0 of 8
Mean interval between rash and weakness (days)	11.9
Diabetes mellitus	4 of 8
Immunocompromise	1 of 8
Post-herpetic neuralgia 4 months after onset	5 of 8
Myotomes corresponding to dermatomes	8 of 8
Disseminated zoster	1 of 8

**Table 2:** Characteristics of 8 patients with segmental zoster paresis

Case	Gender	Age	Interval between rash and weakness	Rash distribution	Weak distribution	Electrodiagnostic localization	Imaging findings	Prognosis	Factors
1	M	47y	2d	Right shoulder and anterolateral arm	Right C5–6 myotomes	a right incomplete C5–6 radiculopathy	–	A fast recovery (3 months)	–
2	F	70y	20d	Right lateral arm and forearm	Right C5–7 myotomes	a right incomplete brachial plexopathy (upper and middle trunk)	Hyperintensity in spinal dorsal horns at C4–5 vertebral levels	No recovery (2.0 years)	Diabetes 5y
3	M	63y	3d	Dorsum and planta of the right foot	Right L5-S1 myotomes	a right L5-S1 radiculoplexopathy	–	No recovery (1.8 years)	Diabetes 3y
4	^a^	80-90y ^a^	22d	Neck first, all body then	Right C8 myotome	a right brachial plexopathy (lower trunk)	–	No recovery (1.9 years)	Diabetes 30y
5	F	87y	14d	Right lateral arm and forearm	Right C6–8 myotomes	a right incomplete C6–8 radiculoplexopathy	increased signal in the C6–8 nerve roots	No recovery (1.0 year)	Diabetes 20y
6	^a^	60-70y ^a^	12d	Right buttocks and lateral calf	Right L5 myotome	a right L5 radiculoplexopathy	–	Partial recovery (1.0 year)	–
7	M	61y	15d	Left thumb, index finger and forearm	Left C6–8 myotomes	a left C7 radiculopathy and median, radial nerves	increased signal in mdian and radial nerves	No recovery (0.5 year)	–
8	F	80y	7d	Right shoulder, anterolateral arm and thumb	Right C5 myotomes	a right C5 radiculopathy	increased signal in the C5 nerve roots	Partial recovery (0.5 year)	–

To protect patient privacy, ^a^ was used

**Fig. 1:**
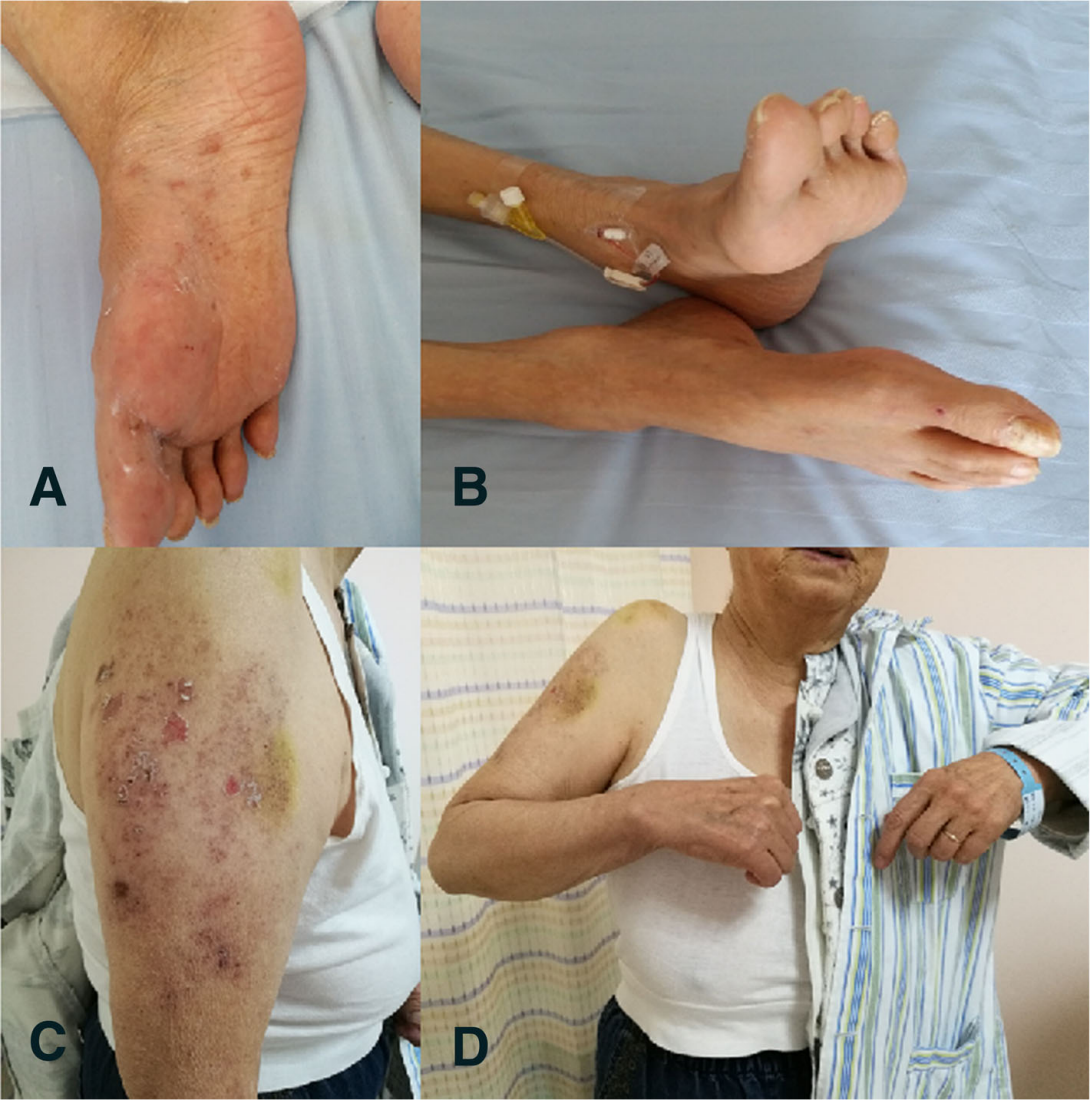
Rash distribution in all patents corresponds to weakness distribution. Scars of a prior herpetic eruption and pigmentation over the dorsum and planta of the right foot were seen in patient 3, who had a foot drop (**a** and **b**). Scars from prior herpetic eruption and pigmentation over the right shoulder and anterolateral arm were seen in patient 8, who could not elevate her shoulder (**c** and **d**)

Rash distribution in all patients corresponded to weakness distribution (Fig. 1). Although the severity of muscle weakness ranged from mild to severe, most were at least moderate. All patients had paresthesia, such as tingling and numbness, corresponding to the distribution of affected myotomes. The deep tendon reflexes were diminished or absent in distribution of affected limbs. In addition, most patients had a certain degree of post-herpetic neuralgia 4 months after onset (5/8).

Four patients had diabetes (case 2, 3, 4 and 5), while 1 patient had a 20-year history of rheumatoid arthritis and use of immunosuppressive medications (case 5). Antiviral drugs, such as acyclovir, were administered to 5 patients (case 1, 2, 5, 6, 7) orally or intravenously within 72 h after the rash appeared, 3 patients after 72 h. In addition, 4 patients received short term use of oral corticosteroids (case 2, 3, 5, 8); nevertheless, no patient experienced immediate pain or weakness relief after treatment.

**Fig. 2:**
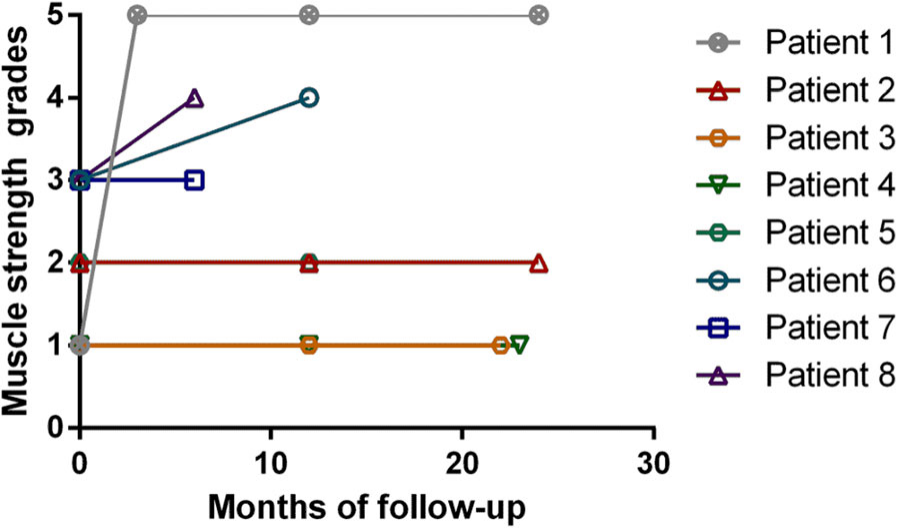
Eight patients had a different prognosis. Patient 1 recovered completely 3 months after symptoms onset. Patient 6 and 8 recovered partially through 1 or 0.5 year respectively. However, the remaining five patients did not recover until 0.5–2.0 years of follow-up

The prognosis was quite different (Fig. 2). Patient 1 recovered completely 3 months after symptoms onset; this patient was placed on oral antiviral medication for 1 week. Patient 6 and 8 recovered partially through 1 or 0.5 year respectively. However, the remaining 5 patients did not recover until 0.5–2.0 years of follow-up. Their muscle strength showed no improvements and one patient’s muscles seemed atrophied (case 7).

### Electrophysiological characteristics

**Fig. 3:**
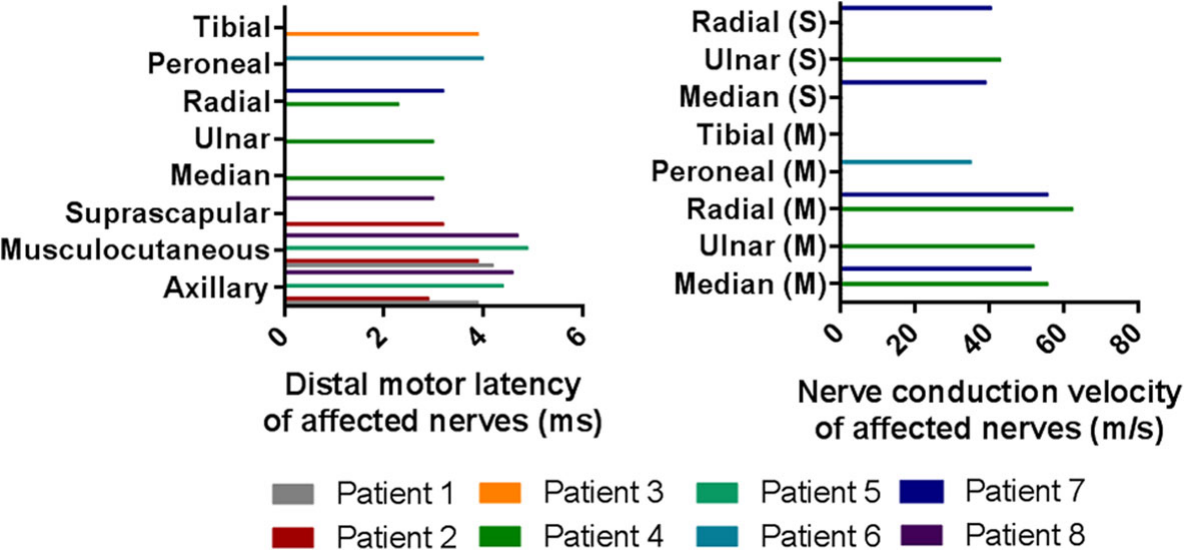
Distal motor latency and nerve conduction velocity of affected nerves. The distal motor latencies were all shorter than 130% of the upper limit of normal. The nerve conduction velocities were all above 35 m/s. M: motor nerve; S: sensory nerve

All electrophysiological examinations were performed no more than 2 weeks after weakness onset. Briefly, nerve conduction studies showed markedly decreased or absent CMAP_S_ and SNAP_S_ amplitudes of affected nerves, compared with the contralateral sides (Table 3). Nerve conduction velocities of affected nerves were normal or mildly decreased, which means they were faster than 75% of the lower limit of normal and above 35 m/s. Distal motor latencies were shorter than 130% of the upper limit of normal. All of these showed axonal lesions, not demyelinating lesions (Fig. 3).

**Table 3:** Nerve conduction studies of patients with segmental zoster paresis

	P1	P2	P3	P4	P5	P6	P7	P8
CMAP of nerves (mV)								
Axillary	12.1(53%↓)	3.5(77%↓)		normal	4.4(51%↓)		normal	6.4(66%↓)
Suprascapular		1.7(84%↓)						3.3(76%↓)
Musculocutaneous	7.3(61%↓)	4.5(52%↓)		normal	3.6(62%↓)		normal	normal
Median	normal	normal		6.2(51%↓)	1.4(83%↓)		normal	normal
Ulnar	normal	normal		5.3(53%↓)	normal		normal	normal
Radial	normal	normal		5.2(55%↓)	normal		4.1(63%↓)	normal
Peroneal			NR			0.7(75%↓)		
Tibial			3.0(79%↓)			normal		
SNAP of nerves (μV)								
Median	normal	NR		normal	NR		2.3(77%↓)	normal
Ulnar	normal	normal		2.8(60%↓)	normal		normal	normal
Radial	normal	normal		normal	NR		4.5(64%↓)	normal
Superficial peroneal			NR			NR		
Sural			NR			normal		

The amplitudes of CMAPs and SNAPs were compared with contralateral side

*CMAP* compound muscle action potential, *SNAP* sensory nerve action potential, *μV* microvolt, *mV* millivolt, *NR* no response

All the patients showed different grades of spontaneous potentials in affected muscles. The average grade was 2+. There was a pattern of decreased recruitment of motor unit action potentials (MUAPs) in weak muscles. The positive sharp waves and fibrillation potentials in affected myotomes and the decreased recruitment of MUAPs suggested ongoing axonal lesions.

According to spontaneous activities in paraspinal muscles and SNAP_S_ amplitudes, 2 among 8 were diagnosed with preganglionic lesions (case 1: C5-C6 radiculopathy; case 8: C5 radiculopathy), 2/8 were characterized as having postganglionic locations (case 2: upper and middle trunk lesions; case 4: lower trunk lesions) and 4 out of 8 were recognized as combined pre- and postganglionic lesions (case 3: L5-S1 radiculoplexopathy; case 5: C6-C8 radiculoplexopathy; case 6: L5 radiculoplexopathy; case 7: C7 radiculopathy and median, radial nerve lesions).

### Imaging characteristics

**Fig. 4:**
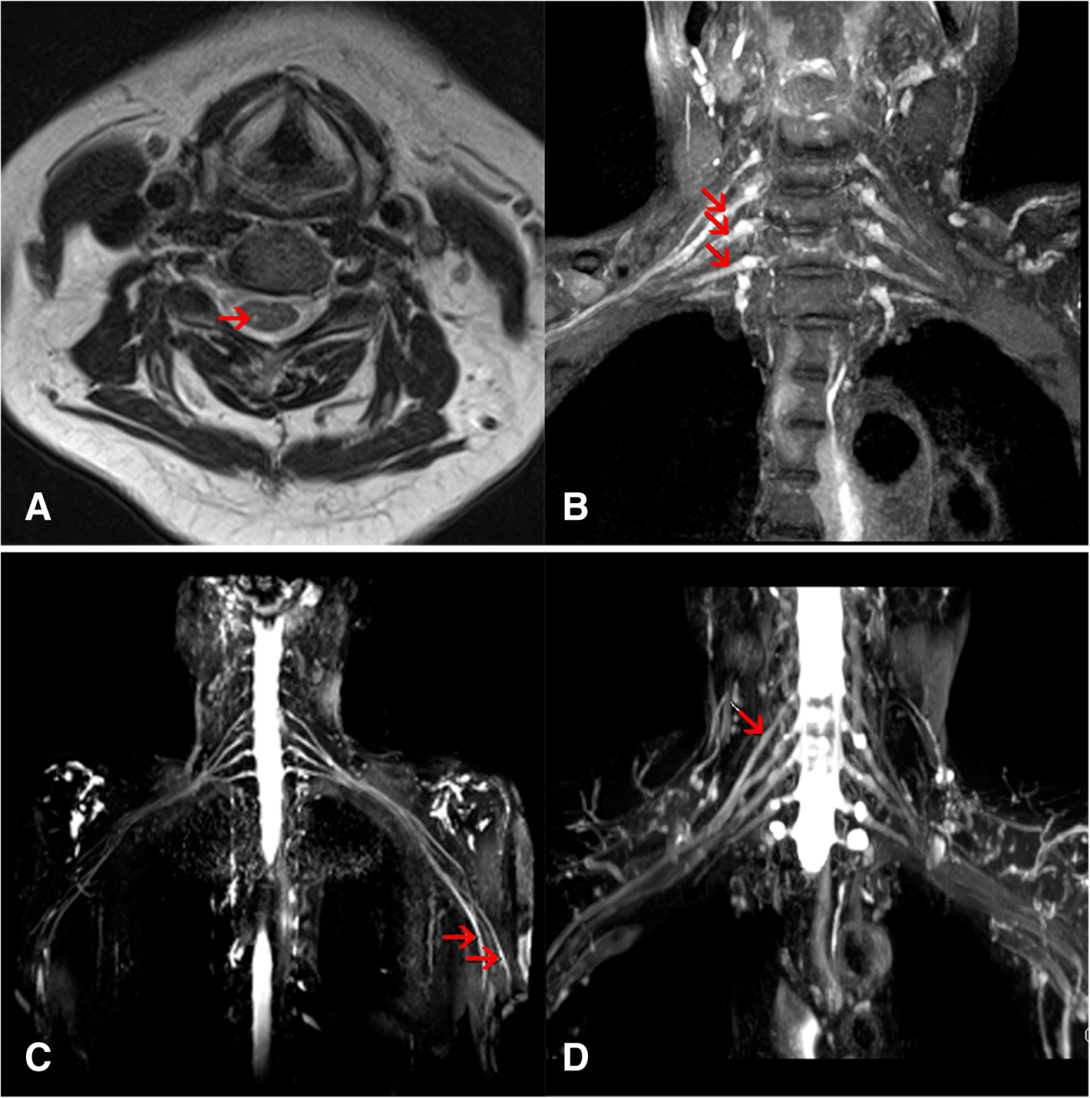
Imaging characteristics of patients with SZP. Axial T2-weighted image showed the unilateral hyperintensity in the dorsal horn of C5 spinal cord in patient 2 (**a**). Brachial plexus magnetic resonance imaging showed hyperintensity of C6–8 nerve roots in patient 5 (**b**), left median and radial nerves in patient 7 (**c**) and C5 nerve roots in patient 8 (**d**)

The MRI examination was performed in 4 out of 8 patients no more than 1 week after electrophysiological testing (Fig. 4). Scans of affected brachial plexus nerves and corresponding spine segments, showed nerve enlargement and enhanced T2 signal intensity, and were in accordance with symptoms (case 5, 7 and 8). In patient 7, median and radial nerve enlargement and T2 hyperintensity were observed, although abnormality of nerve root was not identified. One patient underwent an MRI scanning on cervical spine, not the affected nerves (case 2), and axial T2-weighted image showed hyperintensity in the dorsal horns of C5–6 spinal cord levels, corresponding to her sensory symptoms.

### Descriptive cases

#### Case 1

A 47-year-old man developed severe burning pain and a vesicular eruption in the right shoulder and anterolateral arm. Two days later after the rash, he was not able to elevate his right arm to the shoulder level or bend the forearm at the elbow joint. Additionally, he also presented with numbness in the back of the thumb. Muscle weakness was present in the right deltoid (1/5), infraspinatus (1/5), supraspinatus (1/5) and biceps (2/5), according to the MRC scale. Distal muscle strength was normal. The right biceps reflex was absent. The electrophysiological examination revealed lower amplitude axillary and musculocutaneous CMAPs (12.1 and 7.3 mV, respectively) compared to contralateral sides (25.7 and 18.9 mV, respectively). The sensory nerve conduction studies were normal. Abnormal spontaneous potentials and decreased recruitments of MUAPs were present in the right deltoid, infraspinatus, biceps and C5–6 paraspinal muscles. In conclusion, the electrophysiologic findings were consistent with the incomplete lesions of C5 and C6 nerve roots.

Within 3 months follow-up period, he regained the full arm strength without any treatments. Electrophysiologically, the amplitude axillary and musculocutaneous CMAPs (21.1 and 16.6 mV, respectively) were normal. Abnormal spontaneous activities in muscles innervated by C5 and C6 nerve roots disappeared and many polyphasic MUAPs were observed.

#### Case 5

An 87-year-old woman developed burning pain and vesicular rash over the right lateral arm and forearm. Two weeks after rash, she was not able to elevate her right arm to the shoulder level, bend the forearm at the elbow joint or grip tightly. Moderate to severe weakness of C6–8 myotomes was observed, as well as the hypoesthesia over the C6–7 dermatomes. The biceps reflex was absent. The electrophysiological examination revealed decreased motor ampltitudes of axillary, musculocutaneous and median nerve (4.4, 3.6 and 1.4 mV, respectively) and the absence of median and radial nerve SNAPs. The needle EMG revealed many positive sharp waves in the right deltoid, biceps, extensor digitorum communis, abductor pollicis brevis and C6 paraspinal muscles. These findings were consistent with a right incomplete C6–8 radiculoplexopathy. Brachial plexus MRI showed hyperintensity of right brachial plexus especially at the C6–8 nerve roots level. Consequently, clinical one-year follow-up revealed that the patient was still not able to elevate her right arm to the shoulder level. Also, she presented with numbness of the thumb and post-herpetic neuralgia.

#### Case 7

A 61-year old man developed burning pain and vesicular rash over left thumb, index finger and forearm. Fifteen days after his rash, he noted weakness in his left hand dorsal stretch and grip. There was moderate weakness of muscles in left C6–8 myotomes and hypoesthesia over the thumb. The triceps muscle stretch reflex was absent. The electrophysiological examination revealed decreased amplitude radial CMAPs (4.1 mV) as compared to the contralateral side (11.1 mV) and decreased amplitudes median and radial nerve SNAPs. The needle EMG revealed many positive sharp waves in the left extensor digitorum communis, brachioradialis, abductor pollicis brevis and C7 paraspinal muscles. These findings were consistent with a left median and radial nerve lesions and nerve root lesion. Moreover, brachial plexus MRI showed hyperintensity of distal median and radial nerve. Though, there was no nerve enlargement or T2 hyperintensity of nerve roots. A half-year follow-up revealed that his muscle strength did not recover, and his muscles seemed atrophied. Also, he developed post-herpetic neuralgia.

## Discussion

In the present study, we confirmed that varicella-zoster virus can lead to SZP which is an uncommon complication of a common ailment, including nerve roots, brachial or lumbar plexus, and peripheral nerves lesions. The electrophysiologic and imageological findings can help demonstrate the extent and severity of affected nerves or nerve roots.

The elderly and immunocompromised patients are most susceptible to VZV [1, 9, 19]. Due to the immunosenescence in aged people, HZ infection frequently occurs [1]. It is a high incidence of disease over the age of 40; the highest incidence is found in the age group from 60 to 70. [2]. The average age in our sample was 69 years old.

Yoleri et al. have suggested that zoster paresis preferentially strikes the upper limbs and then the lower limbs [8], while according to other studies, the upper and lower extremities are equally affected [3, 20]. In the present study, upper limbs were involved in 6 out of 8 patients; which was in line with the former results. The weakness in upper limbs most commonly occurs in C5–7 segments, while the weakness in lower extremities most frequently occurs in L1–4 segments [5]. The involvement of C8 myotome is relatively rare [10]. In other words, the distribution of muscle weakness is common in the proximal muscles of the limbs and limb-girdles. In most of our cases, the upper extremities were involved, which was in accordance with this pattern; although in 3 patients the distal muscles were also involved. In 2 cases of affected lower limbs, weakness did not fit this pattern and indicated the distal weakness. Interestingly, right limbs were involved in 7 out of 8 patients, which was in accordance with previous literatures [5–18, 21–27].

Our electrophysiological study manifested low amplitudes of CMAPs or SNAPs together with many spontaneous activities in all patients, thus suggesting motor and sensory axonopathy. In axonal loss lesions, NCS and DML were normal or mildly slow. Generally, NCV was above 35 m/s and DML was shorter than 130% of the upper normal limit in axonal loss lesions. All our results from 8 patients met the criteria. Consequently, peripheral neuropathy caused by HZ with predominant axonopathy could be seen through our cases electrophysiologically. Sachs et al. have reported an electrophysiological study of zoster paresis for 22 months of follow-up, which appeared to be axonopathy and reinnervation [28].

The dorsal root ganglion is the seat of VZV previous to the HZ eruption; therefore, it is no wonder that amplitudes of SNAPs were low, showing sensory neurons or axons damage. According to existing pathologic studies, demyelination, axon degeneration and lymphocyte infiltration can be found in affected nerves, dorsal root ganglions and dorsal horns [29]. These findings were observed in the postmortem examinations long time after HZ infections. Thus, it’s possible that the demyelination found in autopsy is secondary to the axon degeneration induced by HZ infection. Meanwhile, our electrophysiological examinations were performed no more than 2 weeks from the onset of weakness. Axonal degeneration may be the early presentation.

Brachial or lumbosacral plexus was affected in 5 out of 8 patients, suggesting the dorsal root ganglion (the seat of virus prior to its eruption) and ventral roots lesions. Although the precise mechanism of SZP remains unclear, it is easy to understand that the spread of VZV along nerve fibers to ventral root, ventral horn, and distal nerves can lead to corresponding lesions.

Four of our patients underwent MRI examination. MRI is a useful tool for diagnosing SZP. The imaging abnormalities included nerve enlargement and T2 signal hyperintensity of dorsal horn and brachial plexus or peripheral nerves, which were consistent with clinical symptoms and elcetrophysiological findings, although these imaging manifestations are not specific and could be found in peripheral nerve inflammation caused by other diseases such as neurobrucellosis [30]. The imaging abnormalities were not found in 100% of patients, while nerve T2 hyperintensity or nerve enlargement was found in 70% of patients [31]. Our patent 7 did not show imaging abnormalities at roots.

In the literature, the prognosis for SZP is generally favorable, but the return of motor function can be incomplete, while recovery time can significantly vary [10, 32]. Two-thirds of their patients had completely or almost fully recovered within a year while the other one-sixth of patients suffered from permanent weakness of extremities, which usually occurred in muscles like diaphragm, anterior tibial and the hand intrinsic muscles [2]. In our study, anterior tibial was involved in 1 patient (case 3), and hand intrinsic muscles were involved in 3 patients (case 4, 5 and 7). Nevertheless, C5–7 myotome muscles were mainly observed, even though not mentioned previously, in patient 2 who still had a poor recovery over 2.0-year follow-up. These patients had a longer recovery time than the 47-year old man. The other reason may be probably due to the older age (patient 2–7) and diabetes mellitus (patient 2–5) [14, 33].

Our study is limited by a small sample size of this rare complication of HZ and retrospective design. Some patients without clinical zoster paresis were not examined with EMG, so there might be electrophysiological abnormalities in these patients. In addition, some patients were likely to have a longer follow-up for prognostic estimation.

## Conclusions

In summary, the present study highlights clinical, electrophysiological and imaging characteristics of this rare motor involvements of HZ. It is associated with significant limb weakness, obvious nerve axons lesion, and it is localized in nerve roots, plexus or peripheral nerves. The information is crucial for neurologists in order to avoid unnecessary misdiagnosis. Also, SZP should be considered in the differential diagnosis of acute painful motor weakness of limbs. The electrophysiological testing and MRI scan can be useful for confirming the diagnosis of SZP.

## Abbreviations

CMAP_S_: Compound muscle action potentials
DML: Distal motor latency
EMG: Electromyography
HZ: Herpes zoster
MRI: Magnetic resonance imaging
MUAPs: Motor unit action potentials
NCS: Nerve conduction studies
NCV: Nerve conduction velocity
SNAP_S_: Sensory nerve action potentials
SZP: Segmental zoster paresis
VZV: Varicella-zoster virus
